# Pattern of blood pressure distribution and prevalence of hypertension and prehypertension among adults in Northern Ethiopia: disclosing the hidden burden

**DOI:** 10.1186/1471-2261-14-33

**Published:** 2014-03-05

**Authors:** Mekoya D Mengistu

**Affiliations:** 1 Department of Physiology, School of Medicine, Addis Ababa University, Addis Ababa, Ethiopia; 2 Department of Physiology, College of Health Sciences, Mekelle University, Mekelle, Ethiopia

**Keywords:** Blood pressure, Prehypertension, Hypertension

## Abstract

**Background:**

Hypertension is the 3^rd^ cause of death accounting for one in eight deaths worldwide. Hypertension was thought to be rare in Africa, but it is now recognized as one of the most important cerebrovascular diseases contributing to about 40% of these diseases in the continent.

The aims of this study were to describe the pattern of blood pressure distribution among adults, and determine prevalence of prehypertension and hypertension among adults in Northern Ethiopia.

**Method:**

The study was done on a community-based sample of 1183 adults of 697 (58.8%) urban and 486 (41.1%) rural residents using statistical multistage sampling procedures. The study was based on the recent WHO and JNC-7 classification of blood pressure. Multi-item structured questionnaires were also developed to elicit additional information on the subjects.

**Results:**

The overall prevalence of hypertension and prehypertension in the study population was 18.1% and 37.2%, respectively. The prevalence of hypertension positively correlated with body mass index and age in both urban and rural residents (P = 0.001). Sex and age adjusted mean systolic blood pressure (SBP) was statistically higher in urban than in rural population (P = 0.001).

**Conclusion:**

Hypertension was found to have high prevalence in the study region. However, people’s awareness and control of hypertension was found to be very poor. Lack of a clear hypertension prevention guidelines and strategies nationwide can aggravate the impact of cardiovascular diseases.

## Background

Hypertension was thought to be rare in Africa, but it is now recognized as one of the most important causes of cerebrovascular diseases contributing to about 40% of the diseases in the continent [1,2]. According to WHO report, hypertension is the 3^rd^ cause of death accounting for one in eight deaths worldwide [3]. The overall worldwide burden of hypertension in the year 2000 was 26.4% of the adult world population, 34.26% in developed and 65.73% in developing countries [4].

According to the recent guideline of Joint National Commitee on Prevention, Detection, Evaluation, and Treatment of High Blood Pressure (JNC VII), blood pressure in adults aged at least 18 years is categorized as normal; < 120/80, prehypertension; 120-139/80-89, and hypertension; ≥ 140/90 [5]. Currently, prehypertension is also increasing the risk of cardiovascular diseases. Beginning at a SBP/DBP of 115/75 mm Hg, the risk of cardiovascular disease doubles with each increment of 20/10 mm Hg [6]. About 62% of cerebrovascular disease and 49% of ischemic heart disease are attributable to suboptimal blood pressure (systolic > 115 mm Hg) [7].

Hypertension is one of the major factors for high mortality of adults in sub-Saharan Africa [8]. According to the health and health-related indicators of Ministry of Health, hypertension was the seventh leading cause of death in Ethiopia in 2008 [9]. However, there are only few recent reports on the prevalence of hypertension in Ethiopia. Almost all prevalence studies were conducted before two to three decades and indicated that hypertension was not a public health problem. Mostly because of such findings hypertension and other cardiovascular diseases were not given due attention for decades. According to this ‘old’ data, the prevalence of hypertension amongst bank employees in Addis Ababa was 11% with 13% in males and 5% in females [10], 0.40% in the semi-nomadic and 3.15% in the urban Oromo population [11], 16% on patients with cardiovascular disease seen at Tuberculosis Center in Addis Ababa [12], 11.6% on Ethiopian outpatients [13]. The purpose of this study, therefore, was to describe the current pattern of blood pressure distribution, and to determine the prevalence of hypertension and prehypertension among adults in urban and rural residents of Northern Ethiopia.

## Methods

### Study design and setting

Cross–sectional descriptive study was conducted on non-institutionalized civilian adult population of 18 years or older living in Humera (902 Km North of Addis Ababa) representing the rural population and Mekelle (783 Km North of Addis Ababa) representing the urban population of Tigray Region which is one of the four biggest regions of Ethiopia. Tigray Region is located in the Northern part of the country, shares common borders with Eritrea in the north, the State of Afar in the east, the State of Amhara in the south, and the Republic of the Sudan in the west. Data were collected during Aug 2010 – Jan 2011. Blood pressure measurement was made by standard cuff and relevant interviewing procedures were used to elicit medical history, socio-demographic and life style characteristics. Prior to the survey, piloting was conducted in order to standardize measurement procedures and reduce inter-observer bias.

### Blood pressure measurement and anthropometry

Blood pressure was measured from left arm of each subject with a standard cuff as described by WHO [14]. The measurement was made after the subject rested for at least 5 minutes in a seated position and after the arm was comfortably supported in semi-flexion at heart level. Study participants were instructed to refrain from drinking any caffeinated beverage and from smoking during the half hour preceding the measurement. The cuff pressure was inflated 30 mmHg above the level at which the radial pulse disappears, then deflated slowly at the rate of about 2 mm per sec and the readings were recorded to the nearest 2 mmHg. The point at which the Korotkoff sound appears was regarded as systolic blood pressure (SBP) and the point at which the sounds completely disappear was taken as diastolic blood pressure (DBP). The measurement was made twice and the average of two readings of SBP and DBP were used to describe the blood pressure of the subject. In case where two readings differed by over 10 mmHg, the examiner obtained a third reading, and the three measurements were averaged. Subjects were classified in to 3 groups based on their measured blood pressure; normal blood pressure, prehypertension, and hypertension. Normal blood pressure was when SBP < 120 mm Hg and DBP < 80 mm Hg whereas prehypertension when SBP ≥ 120 mm Hg but < 140 mm Hg or DBP ≥ 80 mm Hg but < 90 mm Hg [5,6]. Hypertension was defined as SBP ≥ 140 mm Hg and/or DBP ≥ 90 mm Hg and/or self reported use of antihypertensive medication [5-7,14].

Body weight was measured, to the nearest 0.5 kg, with the subject standing motionless on the weighing scale, feet about 15 cm apart, and weight equally distributed on each leg. Subjects were instructed to wear minimum outerwear and no footwear while their weight is being measured [14]. Height was measured, to the nearest 0.5 cm, with the subject in an erect position against vertical surface and the head positioned so that the top of the external auditory meatus is level with the inferior margin of the body orbit [14]. Body mass index (BMI) was calculated as weight in kilograms divided by height in meters squared [7,8]. The conventional BMI cutoff points were applied to classify the study populations into underweight (BMI < 18.5 kg/m^2^), normal BMI (18.5 kg/m^2^ ≥ BMI < 25 kg/m^2^), over weight or obese (BMI ≥ 25 kg/m^2^) [7].

### Quality control measures

In order to ensure the accuracy, completeness, and comparability of blood pressure and anthropometric variables, and interview responses across the study sites, different quality control measures were incorporated in the study design. All data collectors had a common training program on how to get reproducible measurements of blood pressure, weight, and height.

### Ethical clearance

Ethical approval was obtained from the Mekelle University Ethics Review Board. Written informed consent was obtained from participants after a comprehensive explanation of the purpose and procedure of the study in local language. For participants who do not read and write, verbal agreement was obtained. Subjects incidentally found with hypertensive conditions during the survey were advised to seek medical attention in the nearby health facility and make lifestyle modifications.

### Statistical analysis

Descriptive statistics were used to calculate proportions. Means were calculated with corresponding 95% confidence intervals (C.I). T-test for continuous variables and chi-square statistics for categorical variables were used in the bivariate analysis. The effects of age, sex, and body mass index on blood pressure were investigated by multiple linear regression using SPSS 15 software package (SPSS inc., Chicago, USA). A p-value < 0.05 was taken as statistically significant.

## Results

### Study sample characteristics

Data were collected from 443 (37.4%) males and 740 (62.4%) females aged at least 18 years of which 697 (58.9%) were urban and 486 (41.1%) were rural residents. About 652 (55%) of the study population were in the age group of 18–29 years. Those in the age group of 30–49 were 25.6% (302) and only 225 (19.1%) of the population had a minimum age of 50 years. About 784 (66.4%) of the study population had normal body mass index with 89.2% (1055) of the population had a monthly income of less than 600 Eth birr. Almost 50% of the study group had an educational background of at most elementary level with majority had no formal education. The prevalence of overweight was 28 (12.6%) in urban males and 79 (16.6%) in urban females (p < 0.05). On the other hand the prevalence of overweight was 20 (9.0%) in rural males and 43 (16.2%) in rural females (p < 0.05). However, there was no significant difference in overweight between urban and rural residents (p > 0.05) (Table 1).

**Table 1:** Description of selected sociodemographic characteristics of the study population

		***Urban***	***Rural***	***Total***
***Residence***		697 (58.8%)	486 (41.1%)	1183
***Sex***	Male	222 (31.9%)	221 (45.5%)	443 (37.4%)
	Female	475 (68.1%)	265 (54.5%)	740 (62.4%)
***Age***	18-29	400 (57.6%)	253 (52.1%)	653 (55.3%)
	30-49	156 (22.5%)	146 (30.0%)	302 (25.6%)
	≥ 50	138 (19.9%)	87 (17.9%)	225 (19.1%)
***Education***	No formal education	224 (32.1%)	185 (38.1%)	409 (34.6%)
	Elementary (1–6)	90 (12.9%)	112 (23.0%)	202 (17.1%)
	Junior (7–8)	89 (12.8%)	85 (17.5%)	(174) 14.7%
	High school (9–12)	201 (28.8%)	98 (20.2%)	299 (25.3%)
	College and above	93 (13.3%)	6 (1.2%)	99 (8.4%)
***Monthly income***	< 600birr/month	619 (88.8%)	436 (89.7%)	1055 (89.2%)
	600-1200birr/month	45 (6.5%)	43 (8.8%)	88 (7.4%)
	> 1200birr/month	32 (4.6%)	7 (1.4%)	39 (3.3%)
***Level of physical activity***	Mostly sedentary	30 (4.2%)	19 (3.9%)	49 (4.0%)
	Involved in simple activities	631(87.6%)	410 (83.5%)	1041 (86.0%)
	Involved in sport and heavier physical activities	59 (8.2%)	62 (12.6%)	121 (10.0%)
***Body mass index***	< 18.5	138 (19.9%)	89 (18.3%)	227 (19.2%)
	18.5 ≥ BMI < 25	450 (64.8%)	334 (68.7%)	784 (66.4%)
	≥ 25	106 (15.3%)	63 (13.0%)	169 (14.3%)

### Distribution of Mean systolic and diastolic blood pressure

**Figure 1:**
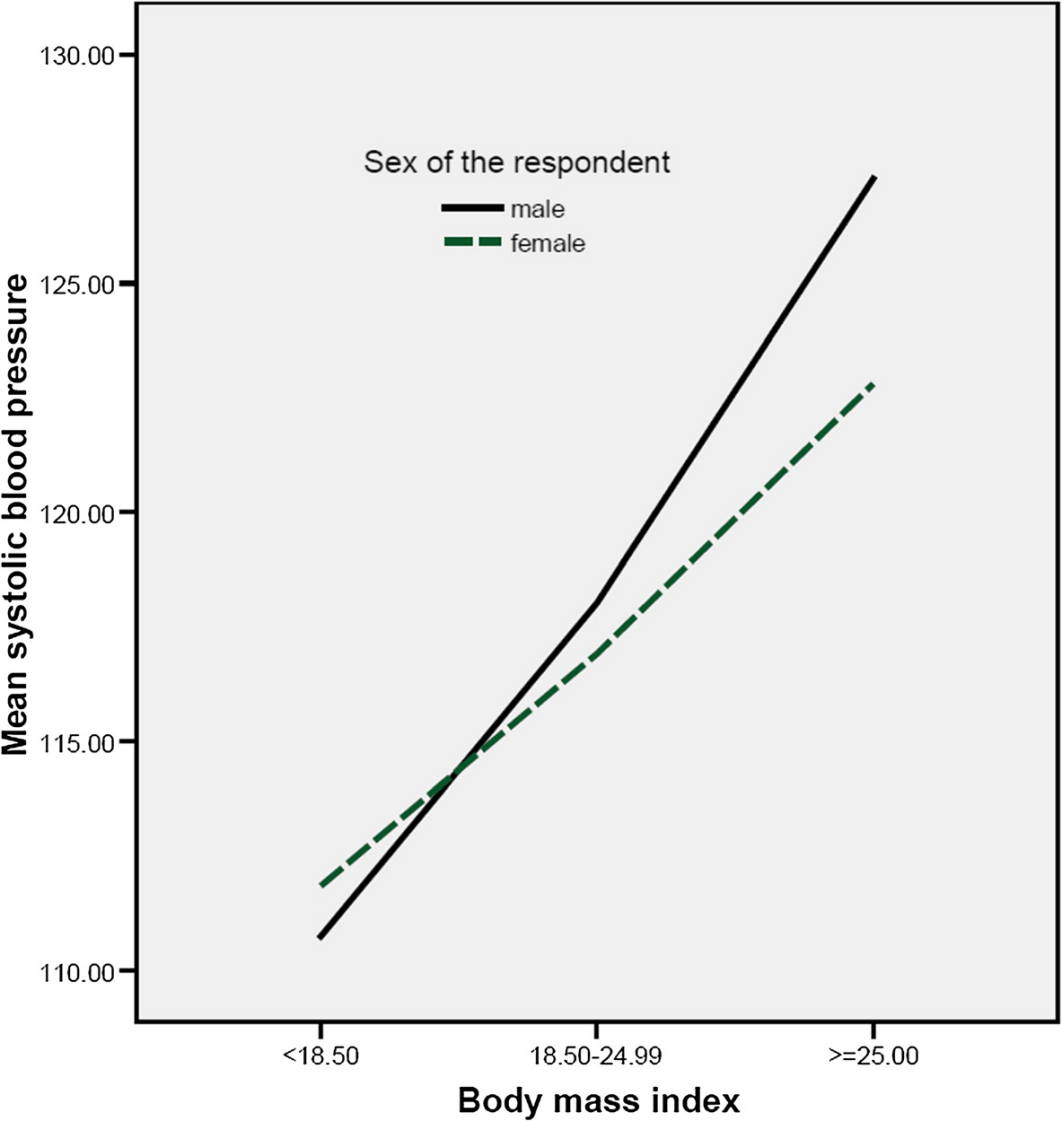
Distribution of mean systolic pressure with body mass index.

**Figure 2:**
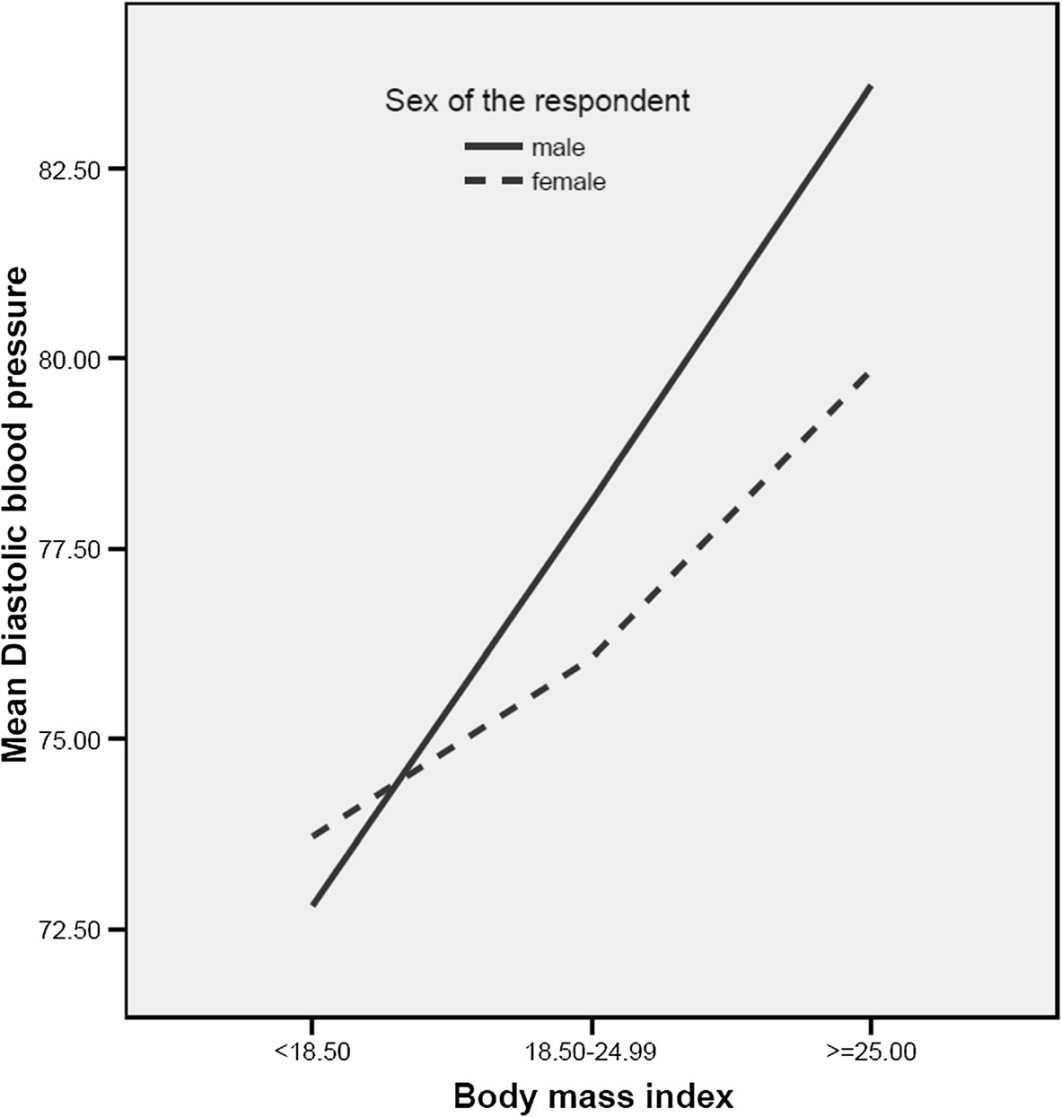
Distribution of mean diastolic pressure with body mass index.

The mean SBP and DBP by sex, age and residence (Urban vs. Rural) are shown in Table 2. Urban males and females had higher mean SBP than their rural counterparts with urban males (120.1+/−18.0, 9% CI: 117.7-121.1) and females (119.0+/−18.3, 9% CI: 117.7-121.1) vs. Rural males (116.2+/−15.5, 95% CI: 114.6-118.0) and females (111.7+/−15.0, 95% CI: 110.2-113.9). However, the mean DBP of urban residents (males 77.5+/−11.0, 95% CI: 75.9-78.9 vs. females 76.3+/−11.1, 95% CI: 75.4-77.4) was comparable with their rural counterpart residents (males 78.2+/−9.2, 95% CI: 76.4-80.0 vs. females 75.6+/−8.9, 95% CI: 72.9-83.8). Sex and age adjusted mean SBP was statistically higher in urban than rural population (P = 0.001). There was positive correlation between SBP with age (P = 0.01). There was also positive correlation between both SBP and DBP with body mass index (P = 0.01) (Figures 1 and 2).

**Table 2:** Comparison of mean SBP and DBP in males and females by age groups for urban and rural population

***Residence***	***Sex***	***Age***	***n (%)***	***SBP+/_SD***	***95% CI***	***DBP+/_SD***	***95% CI***
**Urban**	Female	18–29	268 (22.6%)	114.0+/−13.0	112.5–115.6	74.2+/−8.7	73.2–75.3
		30–49	112 (9.5%)	117.6+/−15.0	115.1–120.9	77.0+/−9.3	75.3–78.8
		≥ 50	100 (8.4%)	134.5+/−25.0	130.3–140.4	81.5+/−16.3	78.3–84.7
		All	480 (40.5%)	119.0+/−18.3	117.7–121.1	76.3+/−11.1	75.4–77.4
	Male	18–29	132 (11.1%)	114.6+/−11.0	112.5–116.3	75.8+/−9.2	74.2–77.4
		30–49	46 (3.9%)	120.6+/−15.1	115.5–124.0	78.6+/−9.9	75.5–81.6
		≥ 50	39 (3.3%)	136.7+/−24.8	127.1–142.9	81.7+/−16.3	76.4–86.9
		All	219 (18.3)	120.1+/−17.1	117.0–121.5	77.5+/−11.0	75.9–78.9
	Total		697 (58.9%)	119.4+/−18.0	118.0–120.7	76.7+/−11.1	75.9–77.6
**Rural**	Female	18–29	152 (12.85%)	107.5+/−12.4	105.6–109.6	74.3+/−8.6	72.9–75.7
		30–49	78 (6.6%)	115.9+/−14.0	114.7–121.9	78.2+/−8.7	76.2–80.2
		≥ 50	39 (3.3%)	120.0+/−19.8	111.5–123.1	75.8+/−9.4	73.0–79.3
		All	269 (22.75%)	111.7+/−15.0	110.2–113.9	75.6+/−8.9	72.9–83.8
	Male	18–29	100 (8.4%)	114.8+/−14.9	114.1–117.8	77.1+/−7.9	75.5–78.7
		30–49	82 (7.0%)	116.9+/−13.8	113.9–119.9	79.6+/−9.6	77.9–82.2
		≥ 50	35 (3.0%)	118.0+/−17.1	109.8–121.9	78.3+/−11.3	72.5–80.3
		All	217 (18.3%)	116.2+/−15.0	114.6–118.0	78.2+/−9.2	76.9–80.0
	Total		486 (41.1′%)	113.9+/−14.3	112.7–115.2	76.8+/−9.1	75.9–77.6
**Total**	Male and female	18–29	654 (55.3%)	112.7+/−13.1	111.7–113.7	75.0+/−8.7	74.3–75.7
		30–49	304 (25.7%)	117.5+/−14.5	115.9–119.2	78.1+/−9.4	77.1–79.2
		≥ 50	225 (19.0%)	128.9+/−23.9	125.7–132.0	79.8+/−14.2	77.9–81.7
	**Male**	**All**	**443 (37.4%)**	**118.1+/−16.4**	**116.6–119.6**	**77.8+/−10.1**	**76.8–78.8**
	**Female**	**All**	**740 (62.6%)**	**116.4+/−17.6**	**115.1–117.6**	**76.1+/−10.4**	**75.4–76.9**
		**All**	**1183 (100%)**	**117.1+/−16.8**	**116.2–118.1**	**76.7+/−10.3**	**76.1–77.3**

### Prevalence of hypertension, prehypertension and awareness of hypertension

The overall prevalence of hypertension and prehypertension in the study population were 18.1% and 37.2%, respectively. In urban residents 22.5% of males and 19.0% of females were found to be hypertensive. However, 19.5% males and 11.5% of females in rural residents were hypertensive. The prevalence rate increased with age in both males and females and in both urban and rural residents (Table 2). Only about 16% of the total hypertensive population (about 20% in urban and about 9% in rural) were aware of having hypertension and about 84% of the hypertensive population were not aware of having high blood pressure. Generally, the prevalence of hypertension was higher among people who educated at most for 6 years (68%) in comparison with those educated for at least high school (23.4%). The prevalence of hypertension positively correlated with body mass index and age in both urban and rural residents (P = 0.001) (Table 3).

**Table 3:** Age and sex adjusted distribution of normal blood pressure, and prevalence of prehypertension and hypertension in urban and rural populations

***Residence***	***Sex***	***Age***	***Normal BP***	***Prehypertension***	***Hypertension***
**Urban**	Male	**18–29**	**71 (53.4%)**	**47 (34.6%)**	**16 (12.0%)**
		**30–49**	**19 (41.3%)**	**13 (28.3%)**	**14 (30.4%)**
		**≥ 50**	**8 (20.5%)**	**14 (35.9%)**	**20 (47.6%)**
		**all**	**98 (44.1%)**	**74 (33.3%)**	**50 (22.5%)**
	Female	**18–29**	**154 (57.9%)**	**87 (32.7%)**	**25 (9.4%)**
		**30–49**	**59 (51.8%)**	**31 (28.2%)**	**22 (19.6%)**
		**≥ 50**	**25 (26.0%)**	**28 (29.2%)**	**43 (44.8%)**
		**all**	**238 (50.2%)**	**146 (30.8%)**	**90 (19.0%)**
	**Total**		**336 (48.3%)**	**220 (31.6%)**	**140 (20.1%)**
**Rural**	Male	**18–29**	**41 (41.0%)**	**46 (46.0%)**	**13 (13.0%)**
		**30–49**	**23 (30.3%)**	**37(48.7%)**	**16 (21.1%)**
		**≥ 50**	**12 (26.7%)**	**19 (42.2%)**	**14 (31.0%)**
		**All**	**76 (34.4%)**	**102 (46.2%)**	**43 (19.5%)**
	Female	**18–29**	**81 (52.9%)**	**62 (40.5%)**	**10 (6.5%)**
		**30–49**	**23 (32.9%)**	**35 (50.0%)**	**12 (17.1%)**
		**≥ 50**	**12 (28.6%)**	**21 (50.0%)**	**9 (21.4%)**
		**All**	**116 (43.8%)**	**118 (44.5%)**	**31 (11.5%)**
	**Total**		**192 (39.5%)**	**220 (45.3%)**	**74 (15.2%)**
**Total**	**Male**	**All**	**174 (39.3%)**	**176 (39.7%)**	**93 (21.0%)**
	**Female**	**All**	**354 (47.9%)**	**264 (35.7%)**	**121 (16.4%)**
	**All**		**528 (44.7%)**	**440 (37.2%)**	**214 (18.1%)**

### Multiple linear regressions

Sex was the leading predictor of mean systolic pressure and diastolic pressure in both rural and urban residents. In the rural residents the mean systolic pressure and diastolic pressure of males were higher than females by 4.5 mm Hg and 2.8 mm Hg, respectively, having controlled for BMI and age (*P* < 0.01 for each). Similarly, in the urban residents the mean systolic pressure and diastolic pressure of males were higher than females by 1.7 mm Hg and 1.04 mm Hg, respectively, having controlled for BMI and age (*P* < 0.01 for each). When rural and urban populations combined, age was the leading predictor of the mean systolic pressure (P < 0.01). However, sex and age were almost equally dominant predictors of the mean diastolic pressure (p < 0.01) as shown in the Table 4.

**Table 4:** Coefficients of multiple linear regressions of SBP and DBP with age, sex and BMI of the study populations

**Blood pressure**	**Predictors**	***Beta***	***p-value***
Mean SBP of the population	Sex	1.897	0.046
	Age	6.757	0.001
	BMI	1.026	0.001
Mean DBP of the population	Sex	1.997	0.001
	Age	1.957	0.001
	BMI	0.656	0.001

## Discussion

As the number of fatalities from cardiovascular diseases decline in west industrial regions, an opposite trend has been observed in the East African region [15]. Hypertension is a strong, consistent, continuous, independent, and etiologically relevant risk factor for cardiovascular-renal diseases [6,16]. The present study showed that the overall prevalence of hypertension in the adult population of the region was 18.1% with 21.0% in males and 16.4% in females. Sex and age adjusted prevalence of hypertension was found to be 22.5% in adult males and 19% in adult females of urban residents. However, 19.5% adult males and 15.2% of adult females in rural residents were hypertensive. This may be partly explained by the relatively high risk factors for hypertension in the urban population such as sedentary lifestyle, cigarette smoking, stress due to high living cost, alcohol, western diet, overweight, and high salt consumption. In the last few decades some of these risk factors such as smoking, alcohol, and chewing chat are very common among males of Mekelle residents.

The prevalence of hypertension was positively correlated with body mass index and age in both urban and rural residents (P = 0.001). Multiple linear regression indicated that sex was the leading predictor of increased mean SBP and DBP in both rural and urban residents. But in the overall population the dominant predictor of increased mean SBP and mean DBP were age (beta = 6.757, p < 0.01) and sex (beta = 1.997, p < 0.01), respectively. It has been clear that aging leads to a multitude of changes in the cardiovascular system including increased vascular stiffness. The age-related increases in blood pressure are mainly attributable to an increase in systolic blood pressure while maintaining or having a slight decrease in a diastolic blood pressure [17].

About 70% of the hypertensive population (62.6% in urban vs. 37.4% in rural) were in stage I and the remaining 30% (77.4% in urban vs. 32.6% in rural) were in stage II. According to JNC 7, stages of hypertension are two with stage I of 140/90-159/99 mm Hg and stage II of ≥ 160/100 mm Hg [5]. This prevalence of hypertension in both urban and rural population of the present study is greater than those reported in earlier studies made decades ago [11-13,18-21]. Since the previous studies [11-13,18-21] were conducted before two to three decades, the current increased living standard in the region with the transforming economy might have caused change in the pattern of salt consumption, obesity, high dietary caloric and cholesterol intake and other risk factors which can be among some of the possible reasons for the variation of hypertension prevalence. The older data depicting a very low prevalence of hypertension [11-13,18-21] were mainly responsible for hypertension and other cardiovascular diseases not given due attention for decades. That also caused some health professionals and the community to be obsessed with the dogmatic notion that our health problems are solely communicable diseases and non-communicable diseases are merely problems of the West [22]. Now there is a compelling need to change the old dogma.

The present result of hypertension prevalence in Northern Ethiopian population (18.1%) is higher than the 13.2% of Southwest Ethiopia [2] but lower than the 28.9-31.5% in Addis Ababa, capital city of Ethiopia [23]. It is also lower than most of the neighboring East African populations such as 18.2%-27% in Sudan [24,25], 30% in Tanzania [26], 30.5% in Uganda [27], 32.6% in Kenya [28]. Nonetheless, there is a growing pattern of hypertension in the region from time to time. Furthermore, among the total hypertensive population, more than 80% of the population was not aware of having high blood pressure (SBP ≥ 140 and or DBP ≥ 90 mm Hg). It is in agreement with the study in Southwest Ethiopia where 84.5% had high BP at the time of the study [2]. This poor level of awareness coupled with high prevalence of hypertension necessitates the development of appropriate and cost effective strategies for primary prevention and treatment of hypertension.

Different studies have shown that prehypertension is also increasing the risk of cardiovascular diseases [6,7]. Beginning at a SBP/DBP of 115/75 mm Hg, the risk of cardiovascular disease doubles with each increment of 20/10 mm Hg [6]. According to classification criteria of JNC 7, the overall prevalence of prehypertension in the present study was 37.2% (33.3% in urban males and 30.8% in urban females vs. 46.2% in rural males and 44.5% in rural females, P < 0.001). This is, therefore, important to firmly recommend practicing of life style modifications for prehypertensive population in order to reduce the risk of developing hypertension in the near future. The prevalence of prehypertension is more in rural than urban residents (45.3% in rural vs. 31.6% in urban, P < 0.001).

The overall mean SBP was higher in urban residents (119.4+/−18.0) than in rural residents (113.9+/−14.3) with P < 0.01. This might be partly because rural residents have low rate of alcohol consumption, less western diet, low salt consumption, lean body build, low level of blood lipid, and engaged in physical activities. However, the mean DBP of urban and rural residents were comparable (P > 0.05). The mean SBP of males was higher than the female counterparts in both rural and urban residents (P < 0.001). The mean SBP correlated positively with both age and BMI in both urban and rural residents (P = 0.01). This is consistent with previous studies done in Ethiopia [7,12,13,20,21]. In the overall study population, the mean DBP of males was also superior to that of females (P < 0.05). This is in agreement with the study done by [7]. The mean DBP of males was higher than that of females in urban residences (P < 0.05) and showed increased trend with age (P < 0.05). However, the mean DBP of males and females in rural residents were insignificantly different and showed no increasing trend with age. This is similar to the studies conducted in 8 rural north-western Ethiopian communities [20] and also to West African population [8].

This study has some limitations. Since inclusion of participants was made based on voluntary participation, this might influence the results. In addition, the age of the study participants generated by questioner may not be accurate as most of the study participants were illiterate and there was no recorded biodata system. Hence this might also influence the results of the study. Furthermore, the study did not employ hematological lipid profiles.

## Conclusion

Hypertension has high prevalence in both urban and rural residents of the region and hence is a public health concern. However, the people’s awareness and control of hypertension was found to be very poor. Therefore, the dogmatic notion that ‘our health problems are solely communicable diseases and non-communicable diseases are merely problems of the West’ must be changed. Lack of a clear hypertension prevention guidelines and strategies nationwide can aggravate the impact of cardiovascular diseases. Hence this is high time to design appropriate control strategies and prevention guidelines involving all stakeholders from government to community levels. The prevalence of prehypertension is also very high in the study area. It is also important to firmly recommend practicing of life style modifications for prehypertensive population in order to reduce the risk of developing hypertension in the near future. In the study population, the rise in the blood pressure correlated with BMI. This requires life style modification to maintain desirable weight. Consumption of diet rich in fruits and vegetables, rich in low-fat dairy products, and reduced in saturated fat and cholesterol are recommended. Finally, further study to uncover the hidden burden of hypertension nationwide is recommended.

## Competing interests

The authors declare that they have no competing interests.

## Pre-publication history

The pre-publication history for this paper can be accessed here:

http://www.biomedcentral.com/1471-2261/14/33/prepub
